# Crystal Gold.—The Manner of Using It and Its Conditions

**Published:** 1855-01

**Authors:** J. Taft


### CRYSTAL GOLD.—THE MANNER OF USING IT AND ITS CONDITIONS.

BY J. TAFT, D. D. S.

In the difficult and intricate operation of filling teeth much depends upon the manner of using the material employed; and very much upon the *condition* at the time of using it. These two points require some consideration at the present time.

There has been a great variety of methods employed in the use of gold foil for filling teeth. It has been used in the *twist* or rolled strip, and the plain strip of three to six thicknesses; the round pellet or ball, rolled between the fingers, without regard to the regularity of the portions composing it; also the small blocks cut from thick folds of foil, and round blocks, either cut from round rolls, or formed by rolling strips upon a fine steel instrument. Each of these forms was introduced in a manner that seemed best adapted to the case. Doubtless, all are acquainted with the manner of introducing and forming each of these into fillings.

The principle design now is, to refer to the manner of forming *Crystal Gold* into fillings.

The portions composing a filling should be introduced as large as will freely enter the cavity, and then set in place, with a large square pointed instrument, serrated upon the end. Only pressure enough to set the piece in place, should be made with this instrument; and then the sharp pointed instrument, with an abrupt bend, should be used for condensing, going over the surface of the filling sufficiently to condense it. And particularly should this instrument be used thoroughly all around the filling next to the wall of the cavity, that the portion of filling next to the walls may be as dense as elsewhere. Some operators seem to have fallen into a mistaken idea that, with the blunt or square pointed instrumeut, they could consolidate equally as well as with the sharp pointed instrument referred to.

By such method of consolidating, two or three difficulties will arise. The blocks will not be uniformly condensed,—that portion next to the instrument will be dense, while that beneath will not be consolidated at all, and the filling will not be dense or consolidated against the walls of the cavity; for the filling will only be consolidated in the direction of the force applied: it will not be pressed laterally, while the instrument is directed to the bottom of the cavity; and it is very certain, if a filling is not well compressed to the walls of the cavity, it will avail but little in arresting caries. When the blunt pointed instrument is used, the portion of filling introduced is not so well prepared for the reception of the succeeding portions as if the sharp pointed condenser had been used.

The amount of pressure requisite for consolidating a filling, will correspond with the point of the condensing instrument. An instrument with a short bend will require the application of far more force to condense a filling, than one with a long and less abrupt bend and shorter joint. An instrument may be so formed that very little pressure is requisite to condense a filling most perfectly. More time is necessary in the use of the instrument so formed, than if it was more abrupt at the point. It is often an important matter to make the least possible pressure upon a tooth in filling.

Gold should be free from deposition of foreign substances upon its surface, in order to make perfect fillings with it. Chemists tell us that platinum very soon becomes so encumbered with foreign substances, that some of its peculiar phenomena cannot be exhibited at all, without first most thoroughly cleansing its surface. Now, the same is true in regard to gold, to a very considerable extent. When impurities of any kind are upon the surface of foil or other preparations of gold, perfect fillings cannot be made with it. Gaseous substances will be condensed with it, or moisture, or impurities of any other kind to which it may be exposed. It is very difficult to keep gold excluded from all things that would thus injure. Gold foil is very liable to be injured in this manner; and other preparations of gold much more so.

*Crystal gold* presents more surface for the lodgment of foreign substances than foil, and becomes correspondingly affected; and hence a very perfect article may, by exposure, become unfit for filling altogether, simply by the lodgment of foreign substances upon the crystals.

This may all be removed by re-annealing, placing the gold upon a piece of charcoal, and with a spirit-lamp and blowpipe, heat the gold to a bright-red. It will, by this process, be perfectly restored, and work as well as when first made, possessing all its original tenacity, and facility for uniting. It will not be injured by frequent annealing; so that its original integrity may thus be maintained. Let those who use crystal gold, anneal it frequently, that they may have it in the best condition for working. It is always well, after receiving a lot, to anneal before using, unless very recently prepared. It will work well for several days, or even weeks after annealed, if not too much exposed.

